# Characterization of the complete mitochondrial genome of convex reef crab *Carpilius convexus* (Forskål, 1775)

**DOI:** 10.1080/23802359.2021.1901618

**Published:** 2021-03-23

**Authors:** Hongtao Liu, Bingshun Li

**Affiliations:** a Ministry of Education, Key Laboratory of Utilization and Conservation for Tropical Marine Bioresources, Hainan Tropical Ocean University, Sanya, PR China; b Hainan Provincial Key Laboratory of Tropical Maricultural Technologies, Hainan Academy of Ocean and Fisheries Sciences, Haikou, PR China

**Keywords:** *Carpilius convexus*, mitochondrial genome, phylogenetic analysis

## Abstract

The complete mitochondrial genome of convex reef crab *Carpilius convexus* was determined and characterized for the first time from the South China Sea. The whole mitogenome is 15,766 bp long and consists of 22 tRNA genes, 2 rRNA genes, 13 protein-coding genes (PCGs), and 1 control region. The nucleotide composition of the mitogenome is significantly biased (A, G, T, and C is 36.91%, 17.94%, 34.95%, and 10.19%, respectively) with A + T contents of 71.86%. All PCGs start with a normal initiation codon ATN and terminate with a standard stop codon except ND1 gene end with TTG. Five microsatellites are identified in *C. convexus* mitogenome sequences. The phylogenetic tree showed that *C. convexus* was first clustered with *Carpilius maculatus*, and strongly supports that the recognition of the Carpiliidae as a monophyletic family.

*Carpilius convexus*, common names red reef crab or convex reef crab, belongs to the family Carpiliidae. It was first described by Peter Forsskål in 1775 as ‘*Cancer convexus,*’ and has sometimes been treated as a variety of the larger species *Carpilius maculatus* (Paulśon 1875). It occurs from the east coast of Africa, through the Red Sea and Arabian Gulf to Hawaii, throughout the warmer regions of the Indo-West Pacific, reaching Japan and Australia (Galil and Vannini 1990). The convex reef crab lives on rocky outcrops or coral reefs, preying on marine bivalve mollusks and dwelling from the lower intertidal to depths of 35 m, where they shelter in crevices or under stones. Despite their size, visually attractive coloration, and economic potential which is taken by divers for the aquarium trade and occasionally collected for food, little is known about their biology to this day. It is considered unfit for consumption because it was found to be mildly toxic, causing body twitching and abdominal muscular spasms in mice lasting 30 min or more in some reports (Holthuis 1968; Garth and Alcala 1977), and recently the brine shrimp lethality assay affirmed that the convex reef crab contains biotoxic compounds (Atanacio 2016). The relationships of carpiliids to other xanthoid crabs and other brachyuran families were studied using mitochondrial gene fragments, but are still less clear (Wetzer et al. 2003; Ahyong et al. 2007; Brösing et al. 2007; Lai et al. 2014).

The samples were collected from the Xisha Islands of Sansha, China (N16°50′24.37″, E112°21′17.70″), and deposited at the marine crustacean specimen room (Hongtao Liu, xmulht@gmail.com) under the voucher number C20191119CC in Qionghai research base of Hainan Academy of Ocean and Fisheries Sciences for reference. The library with an average length of 350 bp was constructed using the NexteraXT DNA Library Preparation Kit, and sequencing was performed on the Illumina Novaseq platform (Total Genomics Solution Limited, SZHT), the 150 bp average length of the generated reads. The whole mitochondrial genome assembled 3.62 G clean reads using the de novo assembler SPAdes version 3.11.0 (Bankevich et al. 2012) and annotated using the MITOS (http://mitos.bioinf.uni-leipzig.de/index.py). A phylogenetic analysis was carried out based on the 13 protein-coding genes (PCGs) encoded by 36 Heterotremata mitogenomes available in GenBank using IQ-TREE version 1.6.12 (Nguyen et al. 2015) by maximum likelihood (ML) method with 1000 bootstrap replicates, the model is mtMet + F+ R5 chosen according to Bayesian information criterion (BIC).

The whole mitogenome of convex reef crab (GenBank Accession No. MT780873) is 15,766 bp in size. The base content was 36.91% A, 17.94% G, 34.95% T, and 10.19% C. The 71.86% of (A + T) showed great preference to AT. It consists of 22 tRNA genes, 2 rRNA genes, 13 PCGs, and one control region. Four PCGs (*ND1, ND4, ND4L*, and *ND5*), eight tRNA genes, two rRNA genes, and the control region were located on the light strand, the others were encoded by the heavy strand.

The 22 tRNA genes in the mitogenome of convex reef crab vary in length from 62 to 74 bp. tRNA-Leu and tRNA-Ser both have two type copies, respectively. The 12S rRNA is 848 bp and is located between tRNA-Val and the control region, and the 16S rRNA is 1374 bp, located between tRNA-Val and tRNA-Leu. Among 13 PCGs, all genes start with a normal initiation codon ATN. Simultaneously, most PCGs terminate with a usual codon TAA or TAG in addition to the ND1 gene using an abnormal stop codon TTG. The control region is 769 bp, located between 12S rRNA and tRNA-Ile. Interestingly, we identified five microsatellites (SSRs) in *C. convexus* mitogenome using MISA. Two (A)_10_ are located in 12S rRNA and ATP8 gene respectively, a (T)_10_ in the ND2 gene, a (TA)_7_, and an (AT)_7_ both in the control region.

The result of the phylogenetic analysis (Figure 1) showed that *C. convexus* was first clustered with *Carpilius maculatus*, and Carpilioidea formed a monophyletic clade with other Heterotremata species. Taken together, the newly sequenced mitochondrial genome of *C. convexus* characterized here should further clarify the phylogenetic relationships of the superfamily in Heterotremata compared with the previous work (Wetzer et al. 2003; Ahyong et al. 2007; Lai et al. 2014).

**Figure 1.**
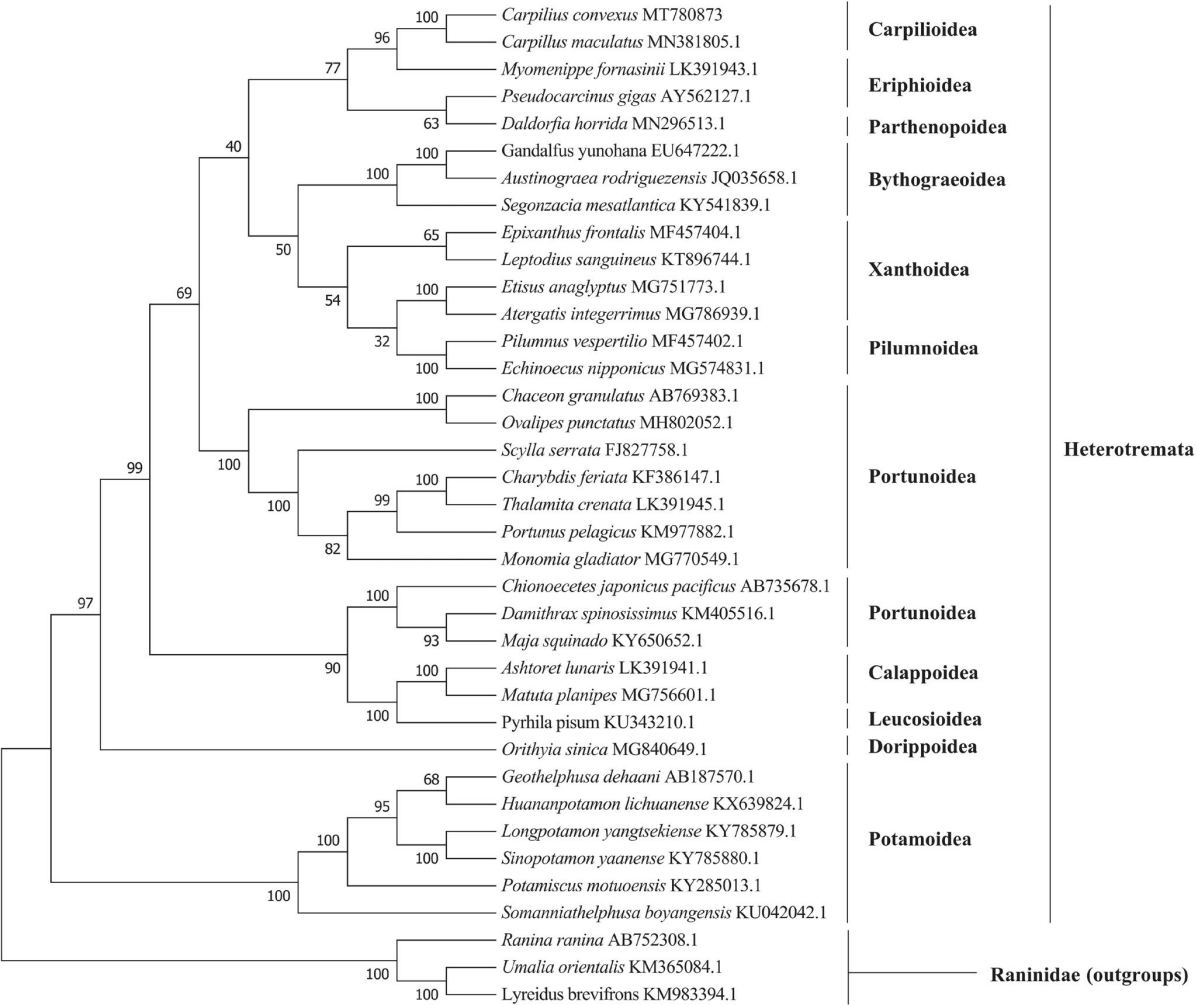
The maximum likelihood tree of *C. convexus* and 36 other species in Heterotremata based on 13 PCGs.

The GenBank accession number for each species is indicated after the scientific name. *Lyreidus brevifrons*, *Umalia orientalis*, and *Ranina ranina* were used as outgroups.

## Data Availability

The genome sequence data that support the findings of this study are openly available in GenBank of NCBI at (https://www.ncbi.nlm.nih.gov/) under the accession no. MT780873. The associated BioProject, SRA, and Bio-Sample numbers are PRJNA699681, SRR13638058, and SAMN17804968 respectively.

## References

[CIT0001] Ahyong ST, Lai JC, Sharkey D, Colgan DJ, Ng PK. 2007. Phylogenetics of the brachyuran crabs (Crustacea: Decapoda): the status of Podotremata based on small subunit nuclear ribosomal RNA. Mol Phylogenet Evol. 45(2):576–586. 17548212 10.1016/j.ympev.2007.03.022

[CIT0002] Atanacio FRB. 2016. Biotoxicological assay of selected Philippine poisonous crab. FASEB J. 30:1192.1194.

[CIT0003] Bankevich A, Nurk S, Antipov D, Gurevich AA, Dvorkin M, Kulikov AS, Lesin VM, Nikolenko SI, Pham S, Prjibelski AD, et al. 2012. SPAdes: a new genome assembly algorithm and its applications to single-cell sequencing. J Comput Biol. 19(5):455–477. 22506599 10.1089/cmb.2012.0021 PMC3342519

[CIT0004] Brösing A, Richter S, Scholtz G. 2007. Phylogenetic analysis of the Brachyura (Crustacea, Decapoda) based on characters of the foregut with establishment of a new taxon. J Zool Syst. 45(1):20–32.

[CIT0005] Galil B, Vannini M. 1990. Research on the coast of Somalia. Xanthidae Trapeziidae Carpiliidae Menippidae (Crustacea Brachyura). Trop Zool. 3(1):21–56.

[CIT0006] Garth JS, Alcala A. 1977. Poisonous crabs of Indo-west pacific coral reefs, with special reference to the genus Demania Laurie. Proceedings, third international coral reef symposium, Rosenstiel school of marine and atmospheric science. Miami (FL): University of Miami; p. 645–651.

[CIT0007] Holthuis L. 1968. Are there poisonous crabs? Crustaceana. 15(2):215–222.

[CIT0008] Lai JC, Thoma BP, Clark PF, Felder DL, Ng PK. 2014. Phylogeny of eriphioid crabs (Brachyura, Eriphioidea) inferred from molecular and morphological studies. Zool Scr. 43(1):52–64.

[CIT0009] Nguyen LT, Schmidt HA, Von Haeseler A, Minh BQ. 2015. IQ-TREE: a fast and effective stochastic algorithm for estimating maximum-likelihood phylogenies. Mol Biol Evol. 32(1):268–274. 25371430 10.1093/molbev/msu300 PMC4271533

[CIT0010] Paulśon OM. 1875. Studies on crustacea of the red sea: with notes regarding other seas. Part I. Podophthalmata and edriophthalmata (Cumacea). SV Kulźhenko. (In Russian).

[CIT0011] Wetzer R, Martin JW, Trautwein SE. 2003. Phylogenetic relationships within the coral crab genus *Carpilius* (Brachyura, Xanthoidea, Carpiliidae) and of the Carpiliidae to other xanthoid crab families based on molecular sequence data. Mol Phylogenet Evol. 27(3):410–421. 12742746 10.1016/s1055-7903(03)00021-6

